# Ultrasound-guided percutaneous sclerotherapy versus surgical resection in the treatment of large hepatic hemangiomas: a retrospective study

**DOI:** 10.1186/s12893-022-01574-3

**Published:** 2022-04-07

**Authors:** Zepeng Lin, Xiaofeng Zhu, Jian Zhou

**Affiliations:** Organ Transplant Center, The First Affiliated Hospital, Sun Yat-Sen University, Guangzhou, China

**Keywords:** Hepatic hemangiomas, Percutaneous sclerotherapy, Surgical resection, Retrospective study, Clinical outcomes

## Abstract

**Background:**

It is no consensus on the best management for patients with large hepatic hemangiomas. This study was designed to evaluate the efficacy and safety of percutaneous sclerotherapy compared to surgical resection for large hepatic hemangiomas.

**Methods:**

A total of 89 patients with large hepatic hemangiomas from single center underwent either percutaneous sclerotherapy (n = 14) or surgical resection (n = 75) as first-line treatment was retrospectively studied, followed up for 9–24 months using ultrasound. Terms of intraoperative and postoperative information, postoperative complications, and treatment effectiveness were compared between the two groups.

**Results:**

Percutaneous sclerotherapy had shorter operative time (*p* < 0.001), less blood loss, lower rate of prophylactic abdominal drainage (97.3% vs. 0%, *p* < 0.001), fewer minor complications (48.0% vs. 7.1%, *p* < 0.01), shorter hospital stay (*p* < 0.001), lower hospital cost (*p* < 0.001), higher Alb level (*p* < 0.001) and lower postoperative clinical index including ALT, AST and WBC (*p* < 0.001 for both) than did surgical resection. The major complications were demonstrated no significant difference between the two groups. In addition, the mean maximum cross-sectional areas of hemangioma dropped from 5044.1 ± 2058.0 mm^2^ to 1924.6 ± 1989.5 mm^2^ (65.2% reduction) during 9–24 months follow-up (*p* < 0.001) in the percutaneous sclerotherapy group, while all patients in the surgical resection group achieved complete response.

**Conclusion:**

Percutaneous sclerotherapy is the preferred method for the treatment of large hepatic hemangioma over surgical resection when compared with the items of postoperative recovery, blood loss, complications, hospital stays, and lower hospital costs. The reduction of the maximum cross-sectional area of hepatic hemangioma in the percutaneous sclerotherapy group is satisfactory.

## Introduction

As the most common benign tumor of liver, hepatic hemangioma has an incidence rate between 0.4 and 20% in autopsied cases [1]. Most hepatic hemangiomas are asymptomatic, small, and require no intervention [2]. However, the large hepatic hemangioma with moderate or severe symptoms or rapidly growing hemangioma may require therapeutic interventions [2–4]. Treatment method for hemangiomas include surgical resection (SR) [5–7], radiofrequency ablation (RFA) [8–10], microwave ablation (MWA) [11–13] and transarterial embolization [14–16]. Traditionally, SR is the most effective treatment. But surgical treatment is often associated with severe trauma, complications, and high risks. MWA and RFA can be performed percutaneously, laparoscopically or by laparotomy, and have been proved to be effective and minimally invasive treatments for patients with large hepatic hemangiomas. However, lengthy MWA or RFA is prone to discomfort and complications such as pain, liver function damage, bleeding, hemolysis, hemoglobinuria, acute kidney injury, tumor rupture and thermal injury to adjacent organs, especially in the treatment of giant hepatic hemangiomas (> 10 cm) [11, 17]. Transarterial embolization is often used to treat acute bleeding in hemangiomas or to reduce the size of hemangiomas before surgery, and can also be used as a single treatment with acceptable outcomes [15, 18]. But to date, there is no consensus on the best management for patients with symptomatic and/or large hemangiomas. The International Society for the Study of Vascular Anomalies has classifieds hepatic hemangiomas as low-flow venous malformations [19]. For subcutaneous low-flow vascular malformations, percutaneous sclerotherapy (PS) has proven to be the standard therapy [20]. Sclerosants can extend drug retention time after being injected in low-flow vascular malformations. Sclerosants retention causes permanent damage to the vascular endothelium of hemangiomas by disrupting cell membranes, leading to sustained vasospasm, tissue ischemia and necrosis [20].

Many studies have explored percutaneous sclerotherapy with bleomycin for the treatment of hepatic hemangioma [21, 22]. However, there is still a lack of research to assess the advantages and disadvantages of ultrasound-guided PS compared with SR for the management of hepatic hemangiomas. For the therapy of hemangioma and vascular malformations, there is no significant difference in efficacy between polidocanol and pingyangmycin sclerotherapy [23]. The purpose of this study was to compared the effectiveness and safety of ultrasound-guided PS using pingyangmycin or lauromacrogol and SR for hepatic hemangiomas.

## Materials and methods

### Patient cohort

This was a single-center retrospective study. We retrieved and reviewed the medical records of patients with large hepatic hemangiomas (large than 5 cm in diameter) who had undergone PS or SR at our hospital from January 2014 to October 2020. All hepatic hemangiomas were diagnosed by two consistent radiologic findings including ultrasound (US), computed tomography (CT) before treatment. The diameters of hepatic hemangiomas were measured on US.

The inclusion criteria for patients undergoing SR are as follows: (1) single or multiple hepatic hemangiomas with a major lesion larger than 5 cm in diameter; (2) obvious symptoms (such as abdominal pain, bleeding, or serious mental burden) or enlarging hemangioma (> 0.5 cm within 12 months); (3) normal liver function (Child–Pugh A level) and normal renal function; no significant irreversible coagulopathy; (4) willingness to undergo surgery.

The inclusion criteria for patients undergoing PS are as follows: (1) single or multiple hepatic hemangiomas with a major lesion larger than 5 cm in diameter; (2) obvious symptoms (such as abdominal pain, bleeding, or serious mental burden) or enlarging hemangioma (> 0.5 cm within 12 months); (3) normal liver function (Child–Pugh A level) and normal renal function; no significant irreversible coagulopathy; (4) haven’t received any other treatment before; (5) have refused for surgery treatment and willing to undergo PS.

The exclusion criteria were listed as follows: (1) patients who did not give consent; (2) history of any prior epigastric surgeries; (3) diagnosed with other types of liver tumors; (4) pregnant woman.

In total, 89 patients with large hepatic hemangiomas were analyzed in this study, of which 14 patients received PS and 75 patients received SR.

### Preprocedural management

Before the specific treatment, all patients underwent preoperative routine tests, including liver and kidney function tests, routine blood tests, coagulation tests, electrocardiogram, abdominal US and abdominal CT examination. Patients over 60 years of age also underwent cardiac US and pulmonary function tests.

### Surgical resection

The surgical resection was carried out in accordance with standard procedures, According to the size and location of the tumor, different types of resection are performed on SR group. Of the 75 patients who underwent surgery, 19 patients underwent hemangioma excision, 1 patient underwent left hemihepatectomy, 8 patients underwent right hemihepatectomy, 8 patients underwent left lateral lobectomy, 2 patients underwent right posterior lobectomy, 2 patients underwent resection of middle lobe of liver, 3 patients underwent irregular hepatectomy, 14 patients underwent laparoscopic hemangioma excision, 14 patients underwent laparoscopic left lateral segmentectomy, 1 patient underwent laparoscopic left hemihepatectomy. 2 patients underwent right hemihepatectomy, 1 patient underwent laparoscopic right posterior lobectomy. In total, 31 patients had 25 cm right subcostal incisions, 12 patients had 25 cm long L-shaped incisions in the right upper abdomen and 32 patients underwent laparoscopic operation through five trocars. Hemangioma excision is generally preferred to liver resection if appropriate. When necessary, the Pringle maneuver was used to control intraoperative blood loss during the operation.

Peripheral blood tests, liver function tests and kidney function tests were performed on the first day after SR. Follow-up ultrasonography or CT examination was performed after the procedure.

### Ultrasound-guided percutaneous sclerotherapy

Pingyangmycin or polidocanol was selected as Sclerosants. To prepare the injection solution, 16 mg pingyangmycin was diluted with 10 mL of physiological saline to a total volume of 10 mL, or polidocanol with the dose of 14–20 mL was injected as original solution. Sclerotherapy was performed under local anesthesia using 10 cc lidocaine 2%, with the patient in a supine position. Hepatic hemangiomas were punctured percutaneously using a 21-G needle (PTC Needle; Hakko Medical Co, Tokyo, Japan) under real-time ultrasound guidance. Then, the prepared polidocanol or pingyangmycin was slowly injected intralesionally over 20–30 s during injection.

After PS, all patients were closely monitored for signs of early complications within 30 min, such as intraperitoneal bleeding and potential sclerosants-induced allergic reactions. If vital signs were stable and ultrasound examination was negative for intra-abdominal free fluid or hematoma patients were return to the ward. Peripheral blood tests, liver and kidney function tests were performed on the first day after SR. Follow-up ultrasonography was performed at 2–8 months after the procedure. A second session of sclerotherapy was planned initially in patients with giant lesions, or with less than 50% shrinkage of lesion volume measured by follow-up imaging examination. The diameters of the treated hemangiomas were measured by US at 9–24 months after the end of the final session. In the end, six patients received two sessions of sclerotherapy, eight patients received a single session of sclerotherapy.

### Criteria for outcomes evaluation

Treatment outcomes were assessed according to postoperative blood tests results, liver and renal function test results, surgical time, intraoperative blood loss, blood transfusion rate, the incidence of complications, hospital stay, hospital costs, technical success rate, and clinical response. Technical success was defined as correct delivery of sclerosants into the hemangioma confirmed by post-sclerotherapy ultrasound. Clinical efficacy is divided into four categories as complete response, marked response, moderate response and mild response. Complete response was defined as reduced scale in hemangioma maximum cross-sectional areas of > 90%. Marked response was defined as a reduced scale in hemangioma maximum cross-sectional areas of from 50 to 90% during follow-ups. Moderate response was defined as a reduced scale in hemangioma maximum cross-sectional areas of from 20 to 50%. Mild response was defined as a reduced scale in hemangioma maximum cross-sectional areas of < 20%.

### Statistical analysis

Statistical analyses were performed using IBM SPSS version 26 for Windows. Parametric continuous data were expressed as means ± standard deviation (SD) and compared using Student’s t-test; Nonparametric continuous data were expressed as mean (range) and compared using Mann–Whitney U test. Categorical parameters were expressed as percentage and compared using the Chi-square test or Fisher’s exact test as appropriate. *p* value < 0.05 was considered statistically significant.

## Results

### Patient characteristics

A total of 89 patients met the inclusion criteria including 75 patients for SR group and 14 for PS. Patient preoperative characteristics are summarized in Table 1. There were no significant differences between the two groups with regard to patient age, sex, body mass index (BMI), hemangioma number, hemangioma size, hemangioma location, white blood cell (WBC), prothrombin time (PT), hemoglobin (Hb), platelet (PLT), liver and renal function indices, such as alanine aminotransferase (ALT), aspartate aminotransferase (AST), total bilirubin (TB), albumin (Alb), serum creatinine (SCr) (*p* > 0.05).

**Table 1:** Baseline characteristics of patients undergoing SR or PS

Characteristics	SR (n = 75)	PS (n = 14)	*p*
Age (years)	44.0 ± 10.0	42.6 ± 8.3	0.635
Sex (male/female)	22 (29.3%)/53(70.7%)	8 (57.1%)/6 (42.9%)	0.087
BMI	22.4 ± 2.8	21.7 ± 2.8	0.395
Number of hemangiomas (solitary/multiple)	34 (45.3%)/41(54.7%)	7 (50%)/7 (50%)	0.748
Hemangioma size (mm)	90.0 (172.0–49.0)	76.0 (152–60)	0.127
Location of hemangioma			0.839
Non-risk areas	65 (86.7%)	13 (92.9%)	
Risk areas^a^	10 (13.3%)	1 (7.1%)	
ALT (U/L)	21.0 (59.0–9.0)	20.5 (41.0–10.0)	0.946
AST (U/L)	24.4 ± 7.7	20.5 ± 5.4	0.076
TB (umol/L)	11.8 (39.0–2.5)	11.1 (20.6–6.0)	0.844
Alb (g/L)	40.8 ± 4.1	40.4 ± 3.8	0.726
WBC (× 10^9^/L)	5.7 (13.0–2.8)	5.6 (8.7–4.1)	0.857
PT (s)	12.1 (15.7–10.1)	11.6 (13.8–10.3)	0.108
Hb (g/L)	125.1 ± 20.6	127.0 ± 22.3	0.753
Plt (10^9^/L)	235.9 ± 65.1	221.0 ± 39.9	0.413
SCr (umol/L)	66.4 (147.0–48.0)	72.0 (107.0–54.0)	0.094

Data are shown as mean ± SD or mean (range)

^a^Hemangiomas in risk areas refer to those located within 5 mm of the diaphragmatic dome, large vessels, or cavity viscera

### Intraoperative and postoperative information

Both PS and SR were completed successfully in all patients. The intraoperative date of the two groups are summarized in Table 2. The operating time in the SR group (192 (505–70) min) was significantly longer than that of the PS group (39 (65–30) min, *p* < 0.001). Moreover, the intraoperative blood loss in the SR group was 494.7 ± 635.1 mL, with a higher intraoperative blood transfusion rate (26.7% vs. 0%, *p* < 0.001), while there is almost no bleeding during PS procedure. Besides, as a routine preventive measure for monitoring bleeding and bile leakage after SR (97.3%), patients in the PS group (0%) were rarely indwelled abdominal drainage tube (*p* < 0.001).

**Table 2:** A comparison of operation data between the SR and PS groups

Factors	SR (n = 75)	PS (n = 14)	*p*^a^
Intraoperative information			
Operation time (min)	192 (505–70)	39 (65–30)	< 0.001
Intraoperative blood loss (mL)	494.7 ± 635.1	–	–
Abdominal drainage (Yes/No)	73 (97.3%)/2 (2.7%)	0 (0%)/14 (100%)	< 0.001
Blood transfusion (Yes/No)	20 (26.7%)/55 (73.3%)	0 (0%)/14 (100%)	0.033
Postoperative information			
Hospital stay time (days)	13 (28–6)	4 (11–2)	< 0.001
Hospital cost (USD)	10,031.0 (93,127.9–4197.9)	1168.0 (1936.1–246.9)	< 0.001

Data are shown as mean ± SD or mean (range)

^a^The *p* values were calculated using Mann–Whitney U test for continuous variables, and Fisher’s exact for categorical variables

The postoperative index was summarized in Table 3. Compared with PS group, The Alb, WBC, ALT and AST levels on day 1 postoperatively was significantly higher in the SR groups (*p* < 0.001). There was no significant difference in the TB, SCr and Plt levels between the two groups on day 1 postoperatively.

**Table 3:** Postoperative clinical index of the patients undergoing SR or PS

Factors	Day 1 post-treatment		*p*^a^
	SR (n = 75)	PS (n = 14)	
Liver function			
ALT (U/L)	176 (1203–26)	18.5 (38–11)	< 0.001
AST (U/L)	201 (1204–41)	25 (32–17)	< 0.001
TB (umol/L)	20.9 (47.2–4.6)	19.2 (29.1–7.8)	0.401
Alb (g/L)	33.8 ± 5.7	40.8 ± 3.6	< 0.001
Renal function			
SCr (umol/L)	59.0 (153–34)	68.5 (110–51)	0.380
Cellular analysis			
WBC (× 10^9^/L)	13.5 (26.9–6.9)	9.8 (17.1–6.8)	< 0.001
Plt (10^9^/L)	174 (391–79)	196 (268–136)	0.157

Data are shown as mean ± SD or mean (range)

^a^The *p* values were calculated using an independent t-test analysis or Mann–Whitney U test

**Fig. 1:**
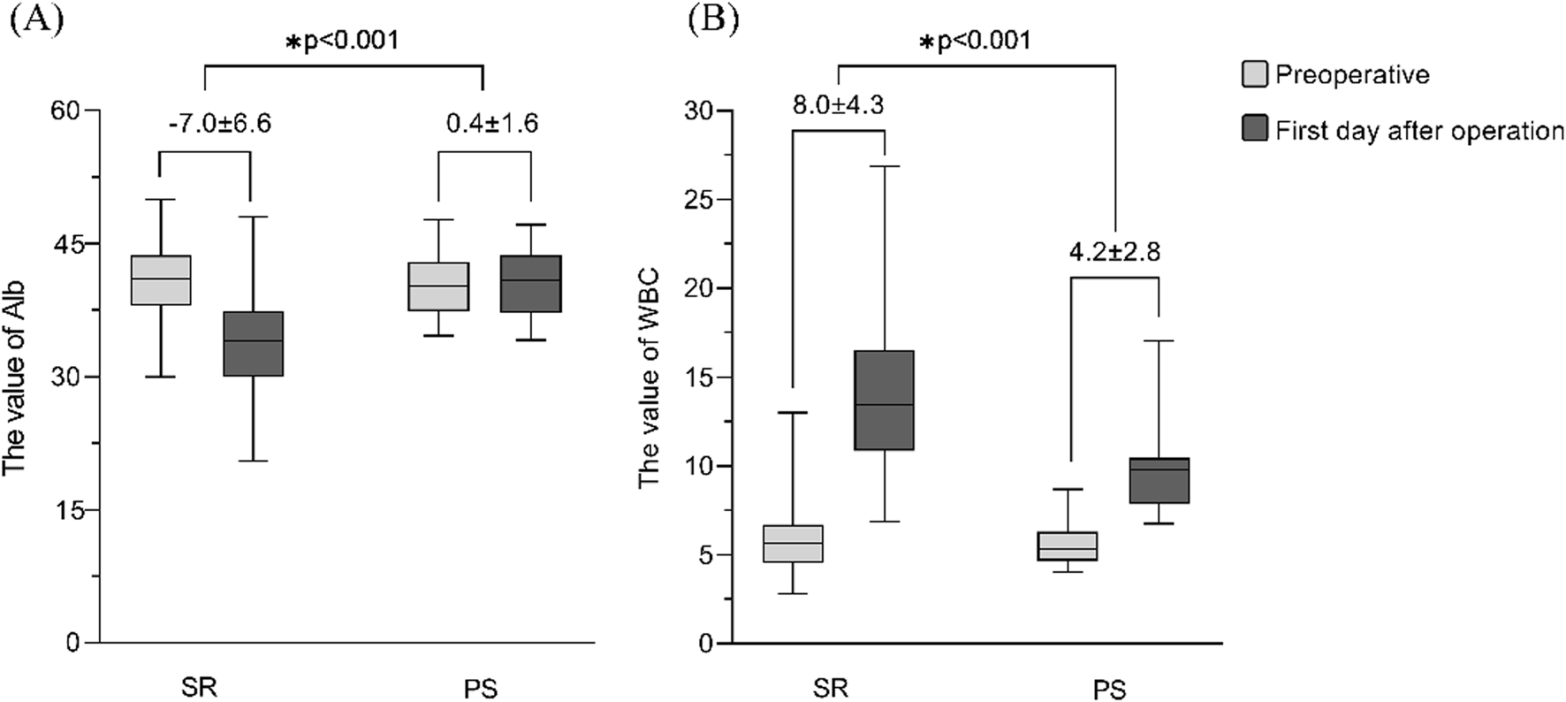
A comparison of preoperative and postoperative biochemical parameters concerning Alb and WBC value between SR and PS group. (value = mean ± SD). (Using an independent t-test and Mann–Whitney U test respectively, the *p *value was calculated to evaluate the changes in factors between SR and PS.) The value marked in the picture presents the changes in factors pre- and post-operation. **A** The Alb value; **B** The WBC value

Compared to preoperative levels, the Alb levels significantly decreased postoperatively in SR group, while there was no significant change in PS groups (Fig. 1). The WBC levels increased postoperatively in both groups, while the increased range in the SR group was significantly higher than in the PS group (*p* < 0.001) (Fig. 1). Furthermore, the ALT and AST levels on day 1 postoperatively increased in SR groups, while there was no significant change in PS groups**.** The increased ALT and AST levels in SR groups continued to decrease to normal levels within 2 weeks (data no show).

What's more, the average hospital stay time (13 (28–6) vs. 4 (11–2) days, *p* < 0.001) for the PS group was significantly less than for the SR group. Patients undergoing SR had significantly higher hospital cost (*p* < 0.001) compared with the PS group, as shown in Table 2.

### Complications between SR and percutaneous sclerotherapy patients

The short-term postoperative complications are shown in Table 4. Postoperative complications are classified by Clavien-Dindo classification [24]. There were no surgery-related deaths in both groups. Minor complications (Clavien Grade I and II) include abdominal pain, fever (≥ 38 ℃), wound infection and intraperitoneal bleeding (needed blood transfusion). There was no significant difference in fever, wound infection and intraperitoneal bleeding complication rates between the groups. However, the incidence of abdominal pain was significantly higher in the SR group than in the PS group (*p* < 0.05). All patients with minor complications recovered within 1–7 days after symptomatic treatment. The overall rate for minor complications in the SR group was significantly higher than in the PS group (*p* < 0.05) (Table 4).

**Table 4:** Complications after treatment

Factors	Clavien Grade	SR (n = 75)	PS (n = 14)	*p*^a^
Minor complications^b^		36 (48%)	2(14.3%)	0.019
Fever^c^	I	19 (25.3%)	1 (7.1%)	0.251
Abdominal pain^d^	I	21 (28%)	0 (0%)	0.034
Wound infection	I	2 (2.7%)	0 (0%)	> 0.99
Intraperitoneal bleeding^e^	II	2 (2.7%)	1 (7.1%)	0.405
Major complications^f^		8 (10.7%)	0 (0%)	0.347
Intraperitoneal bleeding^g^	III	1 (1.3%)	0 (0%)	> 0.99
Bile leakage	III	1 (1.3%)	0 (0%)	> 0.99
Seroperitoneum	III	3 (4%)	0 (0%)	> 0.99
Symptomatic pleural effusion	III	6 (8%)	0 (0%)	0.584

^a^Comparisons were made using Chi-square test or Fisher’s exact test

^b^Minor complications: Clavien Grade I and II

^c^Fever: ≥ 38 °C

^d^Abdominal pain: need analgesic

^e^Intraperitoneal bleeding: received blood transfusion

^f^Major complications: Clavien Grade III, IV and V

^g^Intraperitoneal bleeding: received surgical intervention

Major complications (Clavien Grade III, IV and V) were observed in 8 patients in the SR group and none in PS group. In SR group, there were six patients with symptomatic pleural effusion, three patients with seroperitoneum, one patient with bile leakage and one patients had intraperitoneal bleeding (needed reoperation for hemostasis), including three patients with two types of major complications. The occurrence rate for major complications was higher in the SR group, whereas there was no statistically significant difference in the incidence of major complications between the two groups (Table 4).

### Effectiveness of SR and PS

As shown in Table 5, the first follow-up US results at 9–24 months post-treatment indicate that the SR group (100%) had a significantly higher complete response rate than in the PS group (28.6%) (*p* < 0.001). All of patient in SR group achieve complete cure.

**Table 5:** Postoperative follow-up outcomes

Treatment outcomes	SR (n = 75)	PS (n = 14)	*p*^a^
Complete response^b^	75 (100%)	4 (28.6%)	< 0.001
Incomplete response	0	10 (71.4%)	< 0.001
Marked response^c^	0	6 (42.8%)	
Moderate response^d^	0	2 (14.3%)	
Mild response^e^	0	2 (14.3%)	

^a^The *p* values were calculated using Chi-square test or Fisher’s exact test

^b^The lesion shrinkage percentage ≥ 90%)

^c^The lesion shrinkage percentage between 50 and 90%

^d^The lesion shrinkage percentage between 20 and 50%

^e^The lesion shrinkage percentage < 20%

**Fig. 2:**
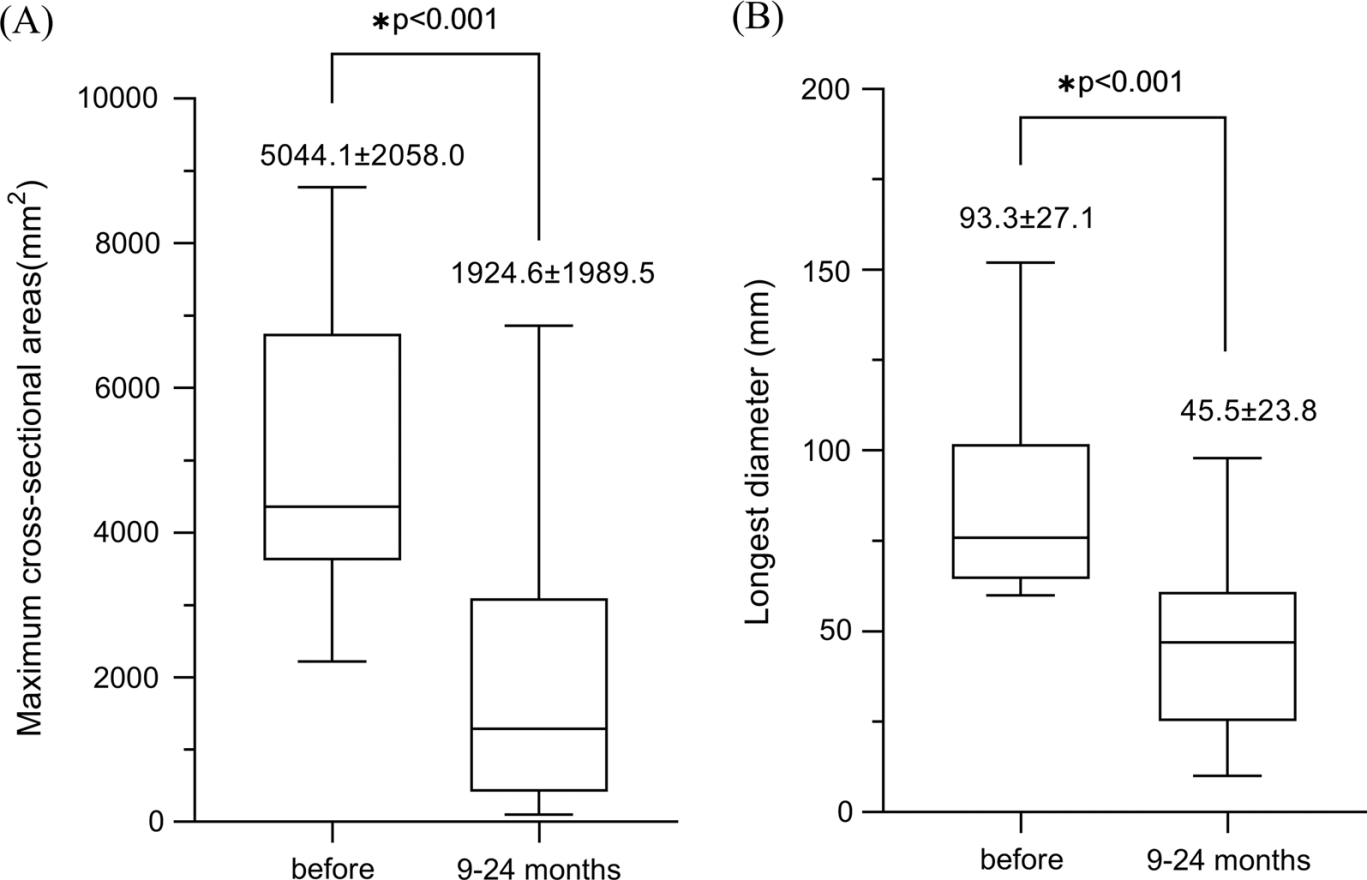
A comparison of maximum cross-sectional areas and longest diameter of lesions before the procedure and 12 months after percutaneous sclerotherapy (value = mean ± SD). The *p* value was calculated using a Mann–Whitney U test. **A** Maximum cross-sectional areas, **B** longest diameter

**Fig. 3:**
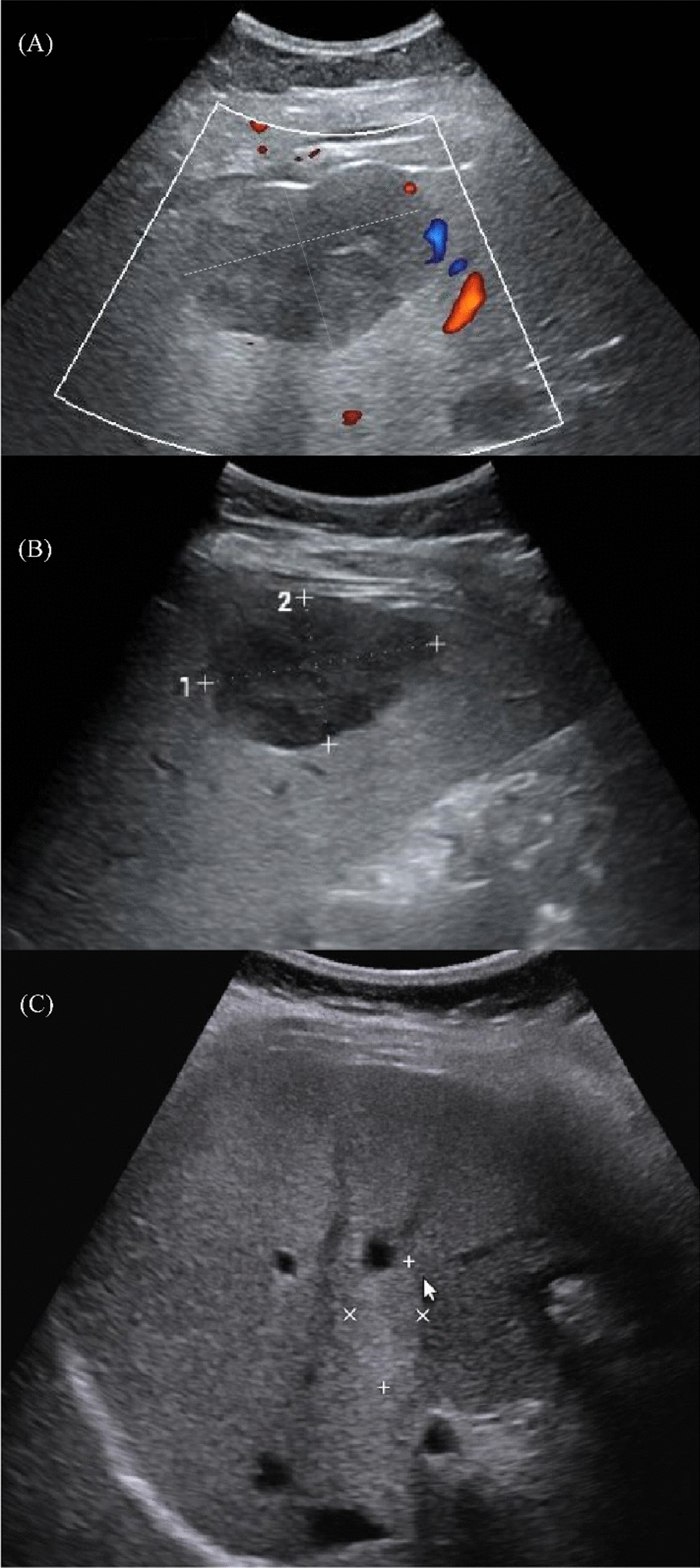
A 41-year-old female with a hepatic hemangioma of 6.0 cm in diameter. **A** Preoperative ultrasound (size: 6.0 cm × 3.7 cm). **B** Ultrasound after 6 months of the first percutaneous sclerotherapy. (size: 5.5 cm × 3.5 cm). **C** Ultrasound after 24 months of the second percutaneous sclerotherapy. (size: 2.7 cm × 1.6 cm)

**Fig. 4:**
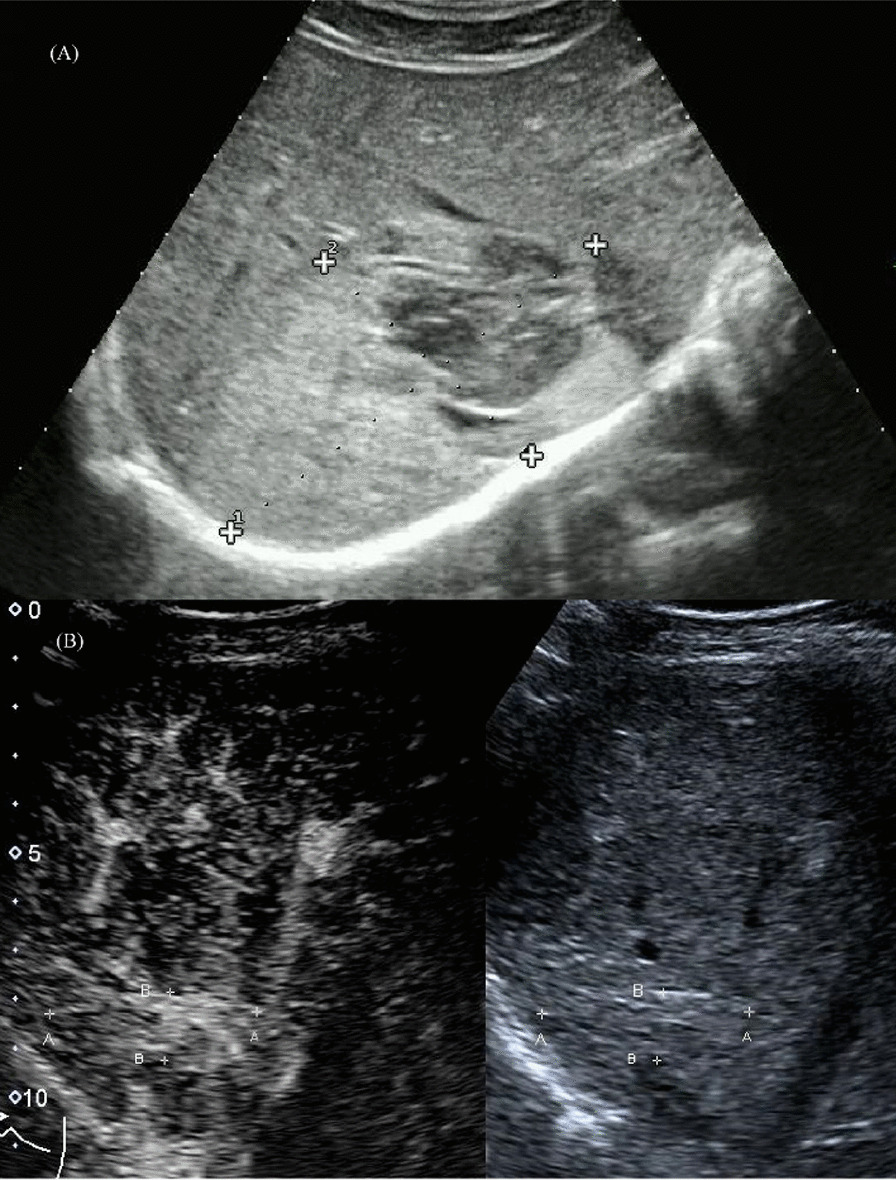
A 33-year-old male with a giant hepatic hemangioma of 10.0 cm in diameter. **A** Preoperative ultrasound (size: 10.0 cm × 6.1 cm). **B** Ultrasound after 12 months of the second percutaneous sclerotherapy. (size: 4.3 cm × 1.4 cm)

In PS group, 10 patients achieved incomplete response, including six patients with marked response with reduction of ≥ 50% in maximum cross-sectional areas of hemangioma. Moderate response was found in 2 patients in the PS group, with reduction range of 20% to 50%. Mild response was found in 2 patients in the PS group with the shrinkage percentage of hemangioma less than 20%. The last follow-up imaging examination showed that the maximum cross-sectional areas of the treated hemangiomas were 1924.6 ± 1989.5 mm^2^ in the PS group, which was significantly smaller than the preoperative level (7545.1 ± 5388.9, *p* < 0.001). Similarly, the longest diameter was 45.5 ± 23.8 mm in the PS group, which was significantly smaller than the preoperative level (93.3 ± 27.1, *p* < 0.001) (Table 6, Figs. 2, 3 and 4). Two clinical ineffective patients were not received further treatment due to the slow growth of their hemangiomas, and their original symptoms had completely resolved.

**Table 6:** Changes in lesion areas and the longest diameter in PS group

Variable	9–24 months Follow-up				*p*^a^
	Before sclerotherapy	After sclerotherapy	Reduction	Reduction (%)	
Lesion areas (mm^2^)	5044.1 ± 2058.0 (8774–2220)	1924.6 ± 1989.5 (6860–100)	3119.5 ± 1788.3 (6404–884)	65.2 ± 28.2 (97–15)	< 0.001
Diameter (mm)	84.4 ± 26.4 (152–60)	45.5 ± 23.8 (98–10)	39.2 ± 23.0 (102–16)	47.3 ± 21.9 (85–14)	< 0.001

Data are shown as mean ± SD (range)

^a^The *p* values were calculated using paired samples t-test

## Discussion

To date, surgical resection is the most common and effective therapy for hepatic hemangioma [3, 4, 6]. Although SR treatment is accurate and effective, it is often associated with high invasive, substantial trauma and high risk. Since the benign nature of hepatic hemangioma, more minimally invasive approaches should be pursued if treatment is needed.

As a minimally invasive procedure, PS is proven to be the standard therapy for low-flow subcutaneous vascular malformations [20]. Under the guidance of imaging devices, sclerosant can be injected into the lesion percutaneously, consequently causing endothelial damage, thrombosis formation, tissue ischemia and ultimately tissue necrosis. Ultrasound guidance is widely used in the process of PS. At present, there are a variety of sclerosants used in the treatment of venous malformations, such as anhydrous alcohol [25], sodium morrhuate [26], pingyangmycin [27, 28], bleomycin [14, 29], and lauromacrogol [30, 31] or polidocanol [23]. In China, the most used agent of local injection therapy for venous malformations is the antitumor agent pingyangmycin, which was similar to bleomycin A5 in chemical structure [32]. Its safety and availability in the treatment of venous malformations as a method of intralesional injection have been confirmed [33]. However, pingyangmycin is only available in China, bleomycin was used as a substitute for pingyangmycin in studies in other countries [14, 21, 29]. Similarly, lauromacrogol is produced in China and has a similar chemical structure to that of polidocanol. For the treatment of hemangiomas, a recent study reported that the efficacy between polidocanol and pingyangmycin is no different [23]. Previously, using a mixture of bleomycin and ethiodized oil as sclerosants, percutaneous sclerotherapy has been reported by many studies and has been suggested as a new and promising treatment method for hepatic hemangioma [21, 22]. However, in terms of efficacy and safety, more information was needed for PS in the treatment of large hepatic hemangioma. This study systematically compared the clinical outcomes of patients with large hepatic hemangioma treated with the SR and PS procedures using pingyangmycin or lauromacrogol.

In general, PS has many advantages in the treatment of large hepatic hemangioma. First of all, the sclerosants were injected into the designated hemangioma area during the process of PS, which causes less damage to the surrounding healthy liver tissue. As a result, there was no increase in the index of liver function tests in PS group 1 day after surgery, while all of the patients who received SR showed a significantly increased liver enzyme index. Second, compared with SR, PS does not require abdominal incision and abdominal drainage, and can be actualized in a shorter operation time. Furthermore, patients who undergone PS have a lower incidence of minor complications, shorter hospitalization stays and lower hospital cost. Third, PS has achieved acceptable therapeutic effects with less invasive. For PS group, complete response was observed in 4 of 14 patients and marked response observed in 6 of 14. Moreover, with the repeatable and less invasive feature, for patients who do not respond well to the first treatment, the second session of PS can be performed.

Just as a coin has two sides, the PS for the treatment of large hepatic hemangiomas has also some disadvantages. For example, the intraperitoneal hemorrhage may occur during puncture. A recent study reported a self-limited intraperitoneal hemorrhage in one patient after percutaneous sclerotherapy with bleomycin [22]. In our study, one patient in SR group experienced self-limited intraperitoneal hemorrhage, while fully recovered after intravenous administration of atropine, hemocoagulase, and intravenous infusions. Nevertheless, a study suggested that the risk of hemorrhage from direct percutaneous puncture of hepatic hemangiomas was low even if large needles are used [34]. Ayoobi et al. performed percutaneous sclerotherapy using 22-gauge needles on 28 participants, none of them had complications of intraperitoneal hemorrhage. Whether the size of puncture needle and the type of sclerosants are related to complications of abdominal bleeding remains to be further studied.

Of course, there are some limitations to our research. First, this is a retrospective study involving the experience of single center, and there might have been selection bias. Second, various surgical resection methods might have affected the incidence of surgical complications. Third, the small sample size and the short period of follow-up may limit the quality of this research. Furthermore, a large sample, prospective randomized controlled studies are required to confirm the findings of this study.

## Conclusion

This study supported the use of ultrasound-guided percutaneous sclerotherapy using pingyangmycin or lauromacrogol as an alternative therapeutic method for large hepatic hemangiomas. Compared with surgical resection, percutaneous sclerotherapy has fewer complications, faster postoperative recovery, shorter hospital stays and lower hospital cost. Whether ultrasound-guided percutaneous sclerotherapy can be used as a first-line treatment option for large hepatic hemangiomas needs to be further confirmed by large sample, multi-center and randomized controlled trials.

## Data Availability

The datasets of the current study are available from the corresponding author upon reasonable request.

## Abbreviations

SR: Surgical resection
PS: Percutaneous sclerotherapy
RFA: Radiofrequency ablation
MWA: Microwave ablation
US: Ultrasound
CT: Computed tomography
BMI: Body Mass Index
ALT: Alanine transaminase
AST: Aspartate aminotransferase
TB: Total bilirubin
Alb: Albumin
WBC: White blood cell
PT: Prothrombin time
Hb: Hemoglobin
Plt: Platelets
SCr: Serum creatinine
USD: United States dollar

## References

[1] Marrero JA, Ahn J, Rajender Reddy K (2014). ACG clinical guideline: the diagnosis and management of focal liver lesions. Am J Gastroenterol.

[2] Schnelldorfer T, Ware AL, Smoot R, Schleck CD, Harmsen WS, Nagorney DM (2010). Management of giant hemangioma of the liver: resection versus observation. J Am Coll Surg.

[3] Leon M, Chavez L, Surani S (2020). Hepatic hemangioma: what internists need to know. World J Gastroenterol.

[4] Abdel Wahab M, El Nakeeb A, Ali MA, Mahdy Y, Shehta A, Abdulrazek M (2018). Surgical management of giant hepatic hemangioma: single center's experience with 144 patients. J Gastrointest Surg.

[5] Yan C, Li BH, Sun XT, Yu DC (2021). Laparoscopic hepatectomy is superior to open procedures for hepatic hemangioma. Hepatobiliary Pancreat Dis Int.

[6] Jinhuan Y, Gang D, Binyao S, Huan M, Bin J (2020). Is laparoscopic hepatectomy suitable for giant hepatic hemangioma larger than 10 cm in diameter?. Surg Endosc.

[7] Liu Q, Liu F, Ding J, Wei Y, Li B (2019). Surgical outcomes and quality of life between laparoscopic and open approach for hepatic hemangioma: a propensity score matching analysis. Medicine (Baltimore).

[8] Qu C, Liu H, Li XQ, Feng K, Ma K (2020). Percutaneous ultrasound-guided 'three-step' radiofrequency ablation for giant hepatic hemangioma (5–15 cm): a safe and effective new technique. Int J Hyperth.

[9] Wen SQ, Wan M, Len KM, Hu QH, Xie XY, Wu Q (2018). Safety and Efficacy of Laparoscopic Radiofrequency Ablation for Hepatic Hemangiomas: A Multicenter Retrospective Study. Ann Hepatol.

[10] Gao J, Ji JS, Ding XM, Ke S, Xin ZH, Ning CM (2016). Laparoscopic radiofrequency ablation for large subcapsular hepatic hemangiomas: technical and clinical outcomes. PLoS ONE.

[11] Wang Z, Tang X, Qi X, Shi Y, Chi J, Li P (2018). Feasibility, safety, and efficacy of ultrasound-guided percutaneous microwave ablation for giant hepatic hemangioma. Int J Hyperth.

[12] Shi Y, Song J, Ding M, Tang X, Wang Z, Chi J (2020). Microwave ablation versus transcatheter arterial embolization for large hepatic hemangiomas: clinical outcomes. Int J Hyperth.

[13] Tang X, Ding M, Lu B, Chi J, Wang T, Shi Y (2019). Outcomes of ultrasound-guided percutaneous microwave ablation versus surgical resection for symptomatic large hepatic hemangiomas. Int J Hyperth.

[14] Akhlaghpoor S, Torkian P, Golzarian J (2018). Transarterial bleomycin-lipiodol embolization (B/LE) for symptomatic giant hepatic hemangioma. Cardiovasc Intervent Radiol.

[15] Torkian P, Li J, Kaufman JA, Jahangiri Y (2021). Effectiveness of transarterial embolization in treatment of symptomatic hepatic hemangiomas: systematic review and meta-analysis. Cardiovasc Intervent Radiol.

[16] Li X, Liu FY, Yuan HJ, Tian XM, Tang J, Ye T (2021). Therapeutic evaluation and management strategy of transarterial embolization for giant liver hemangiomas exceeding 10 cm in diameter. Cardiovasc Intervent Radiol.

[17] Gao J, Fan RF, Yang JY, Cui Y, Ji JS, Ma KS (2017). Radiofrequency ablation for hepatic hemangiomas: a consensus from a Chinese panel of experts. World J Gastroenterol.

[18] Furumaya A, van Rosmalen BV, Takkenberg RB, van Delden OM, Dejong CHC, Verheij J (2019). Transarterial (chemo-)embolization and lipiodolization for hepatic haemangioma. Cardiovasc Intervent Radiol.

[19] Merrow AC, Gupta A, Patel MN, Adams DM (2016). 2014 revised classification of vascular lesions from the International Society for the Study of Vascular Anomalies: radiologic-pathologic update. Radiographics.

[20] Burrows PE, Mason KP (2004). Percutaneous treatment of low flow vascular malformations. J Vasc Interv Radiol.

[21] Ayoobi Yazdi N, Mehrabinejad MM, Dashti H, Pourghorban R, Nassiri Toosi M, Rokni Yazdi H (2021). Percutaneous sclerotherapy with bleomycin and ethiodized oil: a promising treatment in symptomatic giant liver hemangioma. Radiology.

[22] Ayoobi Yazdi N, Dashti H, Batavani N, Borhani A, Shakiba M, Rokni Yazdi H (2018). Percutaneous sclerotherapy for giant symptomatic liver hemangiomas: a pilot study. J Vasc Interv Radiol.

[23] Gao Z, Zhang Y, Li W, Shi C (2018). Effectiveness and safety of polidocanol for the treatment of hemangiomas and vascular malformations: a meta-analysis. Dermatol Ther.

[24] Dindo D, Demartines N, Clavien PA (2004). Classification of surgical complications: a new proposal with evaluation in a cohort of 6336 patients and results of a survey. Ann Surg.

[25] Seruga T, Lucev J, Jevsek M (2015). Treatment of tongue cavernous haemangioma with direct puncture and sclerotization with ethanol. Radiol Oncol.

[26] Schwarcz RM, Ben Simon GJ, Cook T, Goldberg RA (2006). Sclerosing therapy as first line treatment for low flow vascular lesions of the orbit. Am J Ophthalmol.

[27] Zheng JW, Yang XJ, Wang YA, He Y, Ye WM, Zhang ZY (2009). Intralesional injection of Pingyangmycin for vascular malformations in oral and maxillofacial regions: an evaluation of 297 consecutive patients. Oral Oncol.

[28] Bai N, Chen YZ, Fu YJ, Wu P, Zhang WN (2014). A clinical study of pingyangmycin sclerotherapy for venous malformation: an evaluation of 281 consecutive patients. J Clin Pharm Ther.

[29] Horbach SER, Rigter IM, Smitt JHS, Reekers JA, Spuls PI, van der Horst C (2016). Intralesional bleomycin injections for vascular malformations: a systematic review and meta-analysis. Plast Reconstr Surg.

[30] Qu H, Lei X, Hu L, Yin X, Du X, Zhang L (2021). Successful endoscopic sclerotherapy using lauromacrogol injection for laryngopharyngeal hemangioma. Ear Nose Throat J.

[31] Lin PF, Chen FC, Chen JY, Jiang CH (2020). Aesthetic outcome of intralesional injection of lauromacrogol as a single-agent treatment for uncomplicated infantile hemangiomas: a long-term follow-up study. J Dermatol.

[32] Mailloux A, Grenet K, Bruneel A, Bénéteau-Burnat B, Vaubourdolle M, Baudin B (2001). Anticancer drugs induce necrosis of human endothelial cells involving both oncosis and apoptosis. Eur J Cell Biol.

[33] Yuan W, Wang X, Xue L, Zhang F (2021). Clinical evaluation and animal experimental study of different mass concentrations of pingyangmycin in the local injection treatment of lip venous malformation. Ann Transl Med.

[34] Chhieng DC (2004). Fine needle aspiration biopsy of liver - an update. World J Surg Oncol.

